# 3-Eth­oxy-4-hydroxy­benzaldehyde

**DOI:** 10.1107/S1600536808030419

**Published:** 2008-09-24

**Authors:** Yong Li, Xinxi Zhang, Jun Zheng, Xiaoling Wang

**Affiliations:** a Department of Environmental Engineering, Anhui University of Technology, Maanshan 243002, People’s Republic of China; b Analysis and Testing Central Facility, Anhui University of Technology, Maanshan 243002, People’s Republic of China

## Abstract

The title compound (ethyl vanillin), C_9_H_10_O_3_, an important food additive and flavouring agent approved by FAO/WHO, has a vanilla odor four times that of vanillin and shows anti­­mutagenic activity. There are two mol­ecules in the asymmetric unit, each having a planar conformation and an intramolecular O—H⋯O bond. Mol­ecules are connected side-by-side, building infinite ribbons along *c* *via* inter­molecular O—H⋯O hydrogen bonds between the carbonyl and hydroxyl groups. The ribbons are then packed into layers perpendicular to the *a* axis.

## Related literature

For anti-mutagenic activity, see: Ohta *et al.* (1986 ▶). For the synthetic method, see: Gradeff & Murayama (1982 ▶). For related literature, see: Li (2008 ▶).
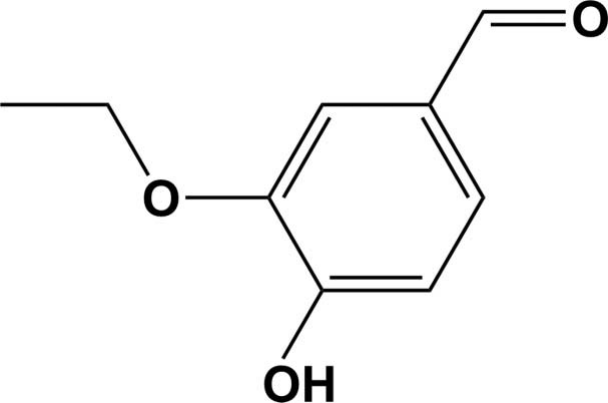


## Experimental

### 

#### Crystal data

C_9_H_10_O_3_
*M* *_r_* = 166.17
Monoclinic, 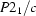

*a* = 13.7352 (6) Å
*b* = 14.4140 (6) Å
*c* = 8.7890 (4) Å
β = 100.742 (3)°
*V* = 1709.55 (13) Å^3^
*Z* = 8
Mo *K*α radiation
μ = 0.10 mm^−1^
*T* = 296 K
0.50 × 0.50 × 0.40 mm

#### Data collection

Bruker SMART CCD area-detector diffractometer
Absorption correction: none
16625 measured reflections
3934 independent reflections
2581 reflections with *I* > 2σ(*I*)
*R*_int_ = 0.025

#### Refinement

*R*[*F* ^2^ > 2σ(*F* ^2^)] = 0.048
*wR*(*F* ^2^) = 0.155
*S* = 1.01
3934 reflections
297 parameters
All H-atom parameters refined
Δρ_max_ = 0.20 e Å^−3^
Δρ_min_ = −0.20 e Å^−3^

### 

Data collection: *SMART* (Bruker, 2002 ▶); cell refinement: *SAINT-Plus* (Bruker, 2003 ▶); data reduction: *SAINT-Plus*; program(s) used to solve structure: *SHELXTL* (Sheldrick, 2008 ▶); program(s) used to refine structure: *SHELXTL*; molecular graphics: *SHELXTL* software used to prepare material for publication: *SHELXTL*.

## Supplementary Material

Crystal structure: contains datablocks global, I. DOI: 10.1107/S1600536808030419/dn2380sup1.cif

Structure factors: contains datablocks I. DOI: 10.1107/S1600536808030419/dn2380Isup2.hkl

Additional supplementary materials:  crystallographic information; 3D view; checkCIF report

## Figures and Tables

**Table 1:** Hydrogen-bond geometry (Å, °)

*D*—H⋯*A*	*D*—H	H⋯*A*	*D*⋯*A*	*D*—H⋯*A*
O2—H1⋯O1^i^	0.79 (3)	2.03 (3)	2.6951 (17)	142 (3)
O2—H1⋯O3	0.79 (3)	2.26 (3)	2.6619 (16)	112 (2)
O12—H14⋯O11^ii^	0.81 (3)	2.02 (3)	2.7117 (19)	143 (3)
O12—H14⋯O13	0.81 (3)	2.26 (3)	2.6554 (18)	111 (2)

Symmetry codes: (i) 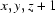; (ii) 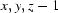.

**Fig. 1.**
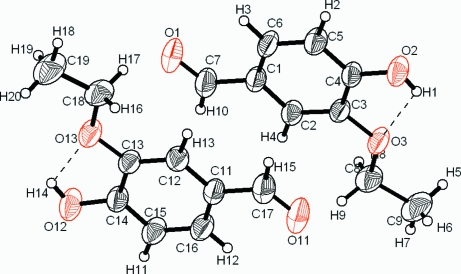
The molecular structure of the title compound with the atom-labeling scheme. Ellipsoids are drawn at the 50% probability level. H atoms are represented as small spheres of arbitrary radii.

**Fig. 2.**
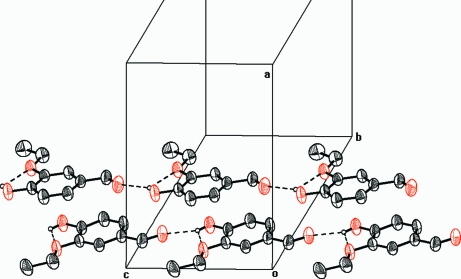
Partial packing view of the title compound, showing one layer of molecules connected by O—H···O hydrogen bonds (dashed lines). H atoms not involved in hydrogen bondings have been removed for clarity.

## supplementary crystallographic information

### Comment

The title compound, an ethyl analogue of vanillin, is an important food additive and flavouring agent approved by FAO/WHO. Its vanilla odor is four times stronger than the flavour of vanillin. Now, it is widely used in food, beverage, cigarette and cosmetics. This synthetic compound was reported to show marked anti-mutagenic activity against mutagenicity induced by 4-nitroquinoline-1-oxide, furylfuramide, captan or methylglyoxal, similar to another report (Li, 2008). It was assumed that the anti-mutagenic activity was due to enhancement of an error-free recombinational repair system (Ohta *et al.*, 1986). But the structure of ethyl vanillin has never been reported. we then  report herein its crystal structure determination (Fig.1). The crystal structure consists of layers of planar molecules linked as one-dimensional chains (Fig. 2).

### Experimental

One of the synthetic methods was reported by literature(Gradeff & Murayama, 1982). The crude title compound commercially available was recrystallized two times from EtOH/water (1:1) solution, and then colourless block crystals were collected after slow evaporation at room temperature.

### Refinement

The structure was solved successfully with direct method. Due to the high quality of diffraction data, *R*(int) = 0.0252, all H atoms were located in a difference map easily and refined isotropically.

### Crystal data

**Table:**

C_9_H_10_O_3_	*F*(000) = 704
*M**_r_* = 166.17	*D*_x_ = 1.291 Mg m^−^^3^
Monoclinic, *P*2_1_/*c*	Mo *K*α radiation, λ = 0.71073 Å
Hall symbol: -P 2ybc	Cell parameters from 17141 reflections
*a* = 13.7352 (6) Å	θ = 2.1–27.6°
*b* = 14.4140 (6) Å	µ = 0.10 mm^−^^1^
*c* = 8.7890 (4) Å	*T* = 296 K
β = 100.742 (3)°	Block, colourless
*V* = 1709.55 (13) Å^3^	0.50 × 0.50 × 0.40 mm
*Z* = 8	

### Data collection

**Table:**

Bruker SMART CCD area-detector diffractometer	2581 reflections with *I* > 2σ(*I*)
Radiation source: sealed tube	*R*_int_ = 0.025
graphite	θ_max_ = 27.6°, θ_min_ = 2.1°
Thin–slice ω scans	*h* = −17→17
16625 measured reflections	*k* = −18→18
3934 independent reflections	*l* = −11→11

### Refinement

**Table:**

Refinement on *F*^2^	Primary atom site location: structure-invariant direct methods
Least-squares matrix: full	Secondary atom site location: difference Fourier map
*R*[*F*^2^ > 2σ(*F*^2^)] = 0.048	Hydrogen site location: inferred from neighbouring sites
*wR*(*F*^2^) = 0.155	All H-atom parameters refined
*S* = 1.02	*w* = 1/[σ^2^(*F*_o_^2^) + (0.0737*P*)^2^ + 0.3872*P*] where *P* = (*F*_o_^2^ + 2*F*_c_^2^)/3
3934 reflections	(Δ/σ)_max_ < 0.001
297 parameters	Δρ_max_ = 0.20 e Å^−^^3^
0 restraints	Δρ_min_ = −0.20 e Å^−^^3^

### Special details

**Table:**

Geometry. Bond distances, angles *etc*. have been calculated using the rounded fractional coordinates. All su's are estimated from the variances of the (full) variance-covariance matrix. The cell e.s.d.'s are taken into account in the estimation of distances, angles and torsion angles
Refinement. Refinement on *F*^2^ for ALL reflections except those flagged by the user for potential systematic errors. Weighted *R*-factors *wR* and all goodnesses of fit *S* are based on *F*^2^, conventional *R*-factors *R* are based on *F*, with *F* set to zero for negative *F*^2^. The observed criterion of *F*^2^ > σ(*F*^2^) is used only for calculating -*R*-factor-obs *etc*. and is not relevant to the choice of reflections for refinement. *R*-factors based on *F*^2^ are statistically about twice as large as those based on *F*, and *R*-factors based on ALL data will be even larger.

### Fractional atomic coordinates and isotropic or equivalent isotropic displacement parameters (Å^2^)

**Table:**

	*x*	*y*	*z*	*U*_iso_*/*U*_eq_	
O1	0.12477 (10)	0.43352 (12)	0.28767 (13)	0.0715 (5)	
O2	0.12710 (11)	0.36758 (9)	1.00143 (14)	0.0604 (5)	
O3	0.12391 (10)	0.54909 (7)	0.94489 (12)	0.0548 (4)	
C1	0.12429 (11)	0.45393 (11)	0.55447 (17)	0.0437 (5)	
C2	0.12350 (12)	0.51971 (11)	0.67073 (17)	0.0448 (5)	
C3	0.12434 (11)	0.49228 (10)	0.82084 (16)	0.0410 (4)	
C4	0.12606 (12)	0.39763 (11)	0.85609 (16)	0.0436 (5)	
C5	0.12674 (14)	0.33230 (12)	0.74025 (19)	0.0542 (6)	
C6	0.12612 (13)	0.36096 (12)	0.59045 (18)	0.0501 (5)	
C7	0.12395 (14)	0.48538 (14)	0.39633 (19)	0.0558 (6)	
C8	0.12357 (18)	0.64675 (12)	0.9186 (2)	0.0601 (7)	
C9	0.1244 (2)	0.69335 (18)	1.0715 (3)	0.0746 (9)	
O11	0.37024 (11)	0.56574 (12)	0.86077 (14)	0.0784 (6)	
O12	0.37991 (12)	0.63363 (10)	0.15095 (15)	0.0686 (5)	
O13	0.37825 (10)	0.45237 (8)	0.20437 (13)	0.0568 (4)	
C11	0.37039 (12)	0.54556 (11)	0.59351 (17)	0.0460 (5)	
C12	0.37298 (12)	0.48002 (12)	0.47729 (18)	0.0467 (5)	
C13	0.37476 (11)	0.50805 (10)	0.32797 (17)	0.0424 (5)	
C14	0.37515 (12)	0.60323 (11)	0.29454 (18)	0.0462 (5)	
C15	0.37110 (14)	0.66787 (12)	0.40983 (19)	0.0549 (6)	
C16	0.36854 (13)	0.63909 (12)	0.5583 (2)	0.0524 (6)	
C17	0.37151 (14)	0.51450 (16)	0.7520 (2)	0.0621 (7)	
C18	0.37312 (17)	0.35399 (12)	0.2254 (2)	0.0591 (7)	
C19	0.3821 (2)	0.30995 (18)	0.0736 (3)	0.0810 (10)	
H1	0.1261 (16)	0.4088 (19)	1.061 (3)	0.080 (7)*	
H2	0.1292 (12)	0.2659 (16)	0.769 (2)	0.070 (6)*	
H3	0.1271 (13)	0.3143 (15)	0.513 (2)	0.064 (5)*	
H4	0.1224 (12)	0.5864 (15)	0.642 (2)	0.065 (6)*	
H5	0.0633 (18)	0.6778 (17)	1.111 (3)	0.097 (8)*	
H6	0.1828 (19)	0.6754 (18)	1.144 (3)	0.101 (8)*	
H7	0.1219 (17)	0.753 (2)	1.052 (3)	0.109 (9)*	
H8	0.0602 (16)	0.6625 (15)	0.843 (3)	0.082 (7)*	
H9	0.1861 (16)	0.6620 (15)	0.876 (2)	0.079 (6)*	
H10	0.1194 (14)	0.5560 (16)	0.378 (2)	0.074 (6)*	
H11	0.3722 (13)	0.7337 (16)	0.381 (2)	0.074 (6)*	
H12	0.3666 (13)	0.6803 (15)	0.638 (2)	0.064 (5)*	
H13	0.3747 (12)	0.4127 (15)	0.507 (2)	0.063 (5)*	
H14	0.3786 (17)	0.592 (2)	0.088 (3)	0.095 (8)*	
H15	0.3753 (13)	0.4451 (15)	0.767 (2)	0.071 (6)*	
H16	0.4291 (15)	0.3369 (14)	0.313 (2)	0.075 (6)*	
H17	0.3088 (15)	0.3405 (14)	0.256 (2)	0.071 (6)*	
H18	0.383 (2)	0.243 (3)	0.097 (4)	0.138 (11)*	
H19	0.325 (2)	0.329 (2)	−0.001 (3)	0.110 (9)*	
H20	0.445 (2)	0.3290 (18)	0.042 (3)	0.105 (9)*	

### Atomic displacement parameters (Å^2^)

**Table:**

	*U*^11^	*U*^22^	*U*^33^	*U*^12^	*U*^13^	*U*^23^
O1	0.0927 (10)	0.0925 (10)	0.0316 (6)	0.0006 (8)	0.0175 (6)	−0.0063 (6)
O2	0.1121 (11)	0.0403 (7)	0.0313 (6)	0.0008 (6)	0.0198 (6)	0.0035 (5)
O3	0.0986 (9)	0.0358 (6)	0.0326 (6)	−0.0012 (6)	0.0188 (5)	−0.0062 (4)
C1	0.0554 (9)	0.0463 (9)	0.0305 (7)	−0.0011 (7)	0.0107 (6)	−0.0016 (6)
C2	0.0664 (10)	0.0357 (8)	0.0333 (8)	−0.0004 (7)	0.0121 (7)	0.0014 (6)
C3	0.0587 (9)	0.0346 (7)	0.0308 (7)	−0.0008 (6)	0.0110 (6)	−0.0041 (6)
C4	0.0660 (10)	0.0364 (8)	0.0297 (7)	0.0002 (7)	0.0125 (6)	0.0016 (6)
C5	0.0917 (13)	0.0337 (8)	0.0389 (8)	0.0001 (8)	0.0169 (8)	−0.0004 (7)
C6	0.0776 (11)	0.0427 (9)	0.0314 (8)	−0.0006 (8)	0.0141 (7)	−0.0081 (7)
C7	0.0722 (11)	0.0635 (12)	0.0327 (8)	−0.0006 (9)	0.0121 (7)	0.0011 (8)
C8	0.0925 (15)	0.0341 (9)	0.0556 (11)	−0.0013 (9)	0.0187 (10)	−0.0071 (8)
C9	0.1020 (19)	0.0521 (12)	0.0708 (15)	0.0004 (12)	0.0193 (13)	−0.0262 (11)
O11	0.1028 (11)	0.0990 (12)	0.0365 (7)	−0.0016 (8)	0.0207 (7)	−0.0071 (7)
O12	0.1270 (12)	0.0454 (7)	0.0374 (7)	0.0021 (7)	0.0254 (7)	0.0043 (6)
O13	0.0963 (9)	0.0380 (6)	0.0384 (6)	0.0026 (6)	0.0188 (6)	−0.0049 (5)
C11	0.0552 (9)	0.0504 (9)	0.0332 (8)	−0.0009 (7)	0.0105 (6)	−0.0028 (7)
C12	0.0628 (10)	0.0391 (9)	0.0393 (8)	−0.0013 (7)	0.0122 (7)	0.0020 (7)
C13	0.0564 (9)	0.0355 (7)	0.0360 (8)	0.0001 (6)	0.0103 (6)	−0.0040 (6)
C14	0.0669 (10)	0.0391 (8)	0.0336 (8)	0.0011 (7)	0.0122 (7)	0.0009 (6)
C15	0.0861 (13)	0.0370 (8)	0.0425 (9)	0.0015 (8)	0.0147 (8)	−0.0047 (7)
C16	0.0704 (11)	0.0473 (9)	0.0408 (9)	0.0010 (8)	0.0134 (8)	−0.0098 (8)
C17	0.0788 (13)	0.0694 (13)	0.0402 (9)	−0.0033 (10)	0.0168 (8)	0.0015 (9)
C18	0.0820 (13)	0.0348 (9)	0.0594 (12)	0.0004 (8)	0.0103 (10)	−0.0076 (8)
C19	0.110 (2)	0.0560 (13)	0.0776 (16)	0.0065 (13)	0.0192 (15)	−0.0263 (12)

### Geometric parameters (Å, °)

**Table:**

O1—C7	1.215 (2)	C8—H8	1.02 (2)
O2—C4	1.3464 (19)	C8—H9	1.02 (2)
O3—C3	1.3644 (17)	C9—H6	0.96 (3)
O3—C8	1.426 (2)	C9—H7	0.88 (3)
O2—H1	0.79 (3)	C9—H5	0.99 (3)
O11—C17	1.211 (2)	C11—C12	1.397 (2)
O12—C14	1.349 (2)	C11—C17	1.461 (2)
O13—C13	1.3587 (19)	C11—C16	1.382 (2)
O13—C18	1.434 (2)	C12—C13	1.378 (2)
O12—H14	0.81 (3)	C13—C14	1.403 (2)
C1—C7	1.461 (2)	C14—C15	1.385 (2)
C1—C6	1.376 (2)	C15—C16	1.376 (2)
C1—C2	1.396 (2)	C18—C19	1.503 (3)
C2—C3	1.375 (2)	C12—H13	1.00 (2)
C3—C4	1.398 (2)	C15—H11	0.98 (2)
C4—C5	1.388 (2)	C16—H12	0.923 (19)
C5—C6	1.378 (2)	C17—H15	1.01 (2)
C8—C9	1.501 (3)	C18—H16	1.012 (19)
C2—H4	0.99 (2)	C18—H17	0.99 (2)
C5—H2	0.99 (2)	C19—H18	0.99 (4)
C6—H3	0.96 (2)	C19—H19	0.96 (3)
C7—H10	1.03 (2)	C19—H20	0.99 (3)
			
O1···O2^i^	2.6951 (17)	C12···H16	2.71 (2)
O2···C6^ii^	3.386 (2)	C12···H17	2.822 (19)
O2···O3	2.6619 (16)	C14···H6^i^	2.92 (3)
O2···O1^iii^	2.6951 (17)	C16···H16^ix^	2.82 (2)
O3···O2	2.6619 (16)	C18···H13	2.612 (18)
O11···O12^iii^	2.7117 (19)	H1···O1^iii^	2.03 (3)
O12···O11^i^	2.7117 (19)	H1···O3	2.26 (3)
O12···C16^iv^	3.372 (2)	H2···H3^ii^	2.44 (3)
O12···O13	2.6554 (18)	H2···O1^ii^	2.88 (2)
O13···O12	2.6554 (18)	H3···O2^v^	2.62 (2)
O1···H3	2.618 (19)	H3···H2^v^	2.44 (3)
O1···H1^i^	2.03 (3)	H3···O1	2.618 (19)
O1···H2^v^	2.88 (2)	H4···H7^iv^	2.45 (4)
O2···H19^iii^	2.78 (3)	H4···H8	2.37 (3)
O2···H5^vi^	2.70 (3)	H4···H9	2.35 (3)
O2···H3^ii^	2.62 (2)	H4···C8	2.579 (18)
O3···H1	2.26 (3)	H4···H10	2.35 (2)
O11···H9	2.91 (2)	H5···O2^vi^	2.70 (3)
O11···H14^iii^	2.02 (3)	H5···C4^vi^	2.88 (3)
O11···H11^vii^	2.90 (2)	H6···O12^iii^	2.76 (3)
O11···H12	2.555 (19)	H6···C14^iii^	2.92 (3)
O12···H12^iv^	2.69 (2)	H7···H4^vii^	2.45 (4)
O12···H6^i^	2.76 (3)	H8···H4	2.37 (3)
O13···H14	2.26 (3)	H8···C2	2.79 (2)
C1···C11	3.586 (2)	H9···H4	2.35 (3)
C2···C11	3.600 (2)	H9···O11	2.91 (2)
C2···C17	3.349 (3)	H9···C2	2.76 (2)
C2···C7^viii^	3.340 (3)	H10···H4	2.35 (2)
C3···C17	3.572 (2)	H11···O11^iv^	2.90 (2)
C3···C7^viii^	3.599 (2)	H11···H12^iv^	2.46 (3)
C6···O2^v^	3.386 (2)	H12···O11	2.555 (19)
C7···C2^viii^	3.340 (3)	H12···O12^vii^	2.69 (2)
C7···C12	3.362 (3)	H12···H11^vii^	2.46 (3)
C7···C3^viii^	3.599 (2)	H13···C18	2.612 (18)
C11···C2	3.600 (2)	H13···H15	2.33 (2)
C11···C1	3.586 (2)	H13···H16	2.27 (3)
C11···C13^ix^	3.525 (2)	H13···H17	2.46 (3)
C12···C12^ix^	3.485 (2)	H13···H18^ii^	2.38 (5)
C12···C7	3.362 (3)	H14···O11^i^	2.02 (3)
C12···C13^ix^	3.571 (2)	H14···O13	2.26 (3)
C13···C12^ix^	3.571 (2)	H15···H13	2.33 (2)
C13···C11^ix^	3.525 (2)	H16···C12	2.71 (2)
C16···O12^vii^	3.372 (2)	H16···H13	2.27 (3)
C17···C3	3.572 (2)	H16···C16^ix^	2.82 (2)
C17···C2	3.349 (3)	H17···C12	2.822 (19)
C2···H8	2.79 (2)	H17···H13	2.46 (3)
C2···H9	2.76 (2)	H18···H13^v^	2.38 (5)
C4···H19^iii^	2.96 (3)	H19···O2^i^	2.78 (3)
C4···H5^vi^	2.88 (3)	H19···C4^i^	2.96 (3)
C8···H4	2.579 (18)		
			
C3—O3—C8	117.59 (12)	H5—C9—H6	111 (2)
C4—O2—H1	112.7 (19)	C8—C9—H5	110.3 (15)
C13—O13—C18	118.12 (12)	C12—C11—C17	119.54 (16)
C14—O12—H14	113 (2)	C16—C11—C17	120.59 (16)
C6—C1—C7	121.16 (15)	C12—C11—C16	119.86 (14)
C2—C1—C7	119.12 (15)	C11—C12—C13	120.38 (15)
C2—C1—C6	119.72 (14)	O13—C13—C14	114.08 (13)
C1—C2—C3	120.48 (14)	C12—C13—C14	119.18 (14)
C2—C3—C4	119.33 (13)	O13—C13—C12	126.72 (14)
O3—C3—C4	114.26 (12)	O12—C14—C15	118.78 (15)
O3—C3—C2	126.41 (13)	C13—C14—C15	120.14 (14)
O2—C4—C5	118.51 (14)	O12—C14—C13	121.08 (14)
C3—C4—C5	120.10 (13)	C14—C15—C16	120.17 (16)
O2—C4—C3	121.39 (13)	C11—C16—C15	120.24 (16)
C4—C5—C6	119.84 (16)	O11—C17—C11	124.5 (2)
C1—C6—C5	120.53 (15)	O13—C18—C19	106.81 (16)
O1—C7—C1	123.93 (18)	C11—C12—H13	117.8 (10)
O3—C8—C9	107.30 (15)	C13—C12—H13	121.8 (10)
C1—C2—H4	118.2 (10)	C14—C15—H11	117.1 (10)
C3—C2—H4	121.3 (10)	C16—C15—H11	122.7 (10)
C4—C5—H2	118.3 (10)	C11—C16—H12	117.4 (12)
C6—C5—H2	121.9 (10)	C15—C16—H12	122.4 (12)
C1—C6—H3	121.5 (12)	O11—C17—H15	120.6 (10)
C5—C6—H3	118.0 (12)	C11—C17—H15	114.8 (10)
C1—C7—H10	116.4 (10)	O13—C18—H16	106.9 (12)
O1—C7—H10	119.6 (10)	O13—C18—H17	107.3 (12)
O3—C8—H8	107.6 (12)	C19—C18—H16	113.3 (11)
C9—C8—H9	111.4 (11)	C19—C18—H17	112.5 (11)
O3—C8—H9	107.1 (12)	H16—C18—H17	109.6 (15)
C9—C8—H8	110.4 (14)	C18—C19—H18	103 (2)
H8—C8—H9	112.8 (17)	C18—C19—H19	107.0 (17)
C8—C9—H6	109.8 (16)	C18—C19—H20	110.5 (15)
C8—C9—H7	105.7 (17)	H18—C19—H19	113 (2)
H5—C9—H7	107 (2)	H18—C19—H20	111 (2)
H6—C9—H7	113 (2)	H19—C19—H20	112 (2)
			
C8—O3—C3—C4	179.15 (16)	C3—C4—C5—C6	0.3 (3)
C8—O3—C3—C2	−0.7 (3)	O2—C4—C5—C6	−179.73 (17)
C3—O3—C8—C9	−179.32 (17)	C4—C5—C6—C1	−0.4 (3)
C13—O13—C18—C19	−178.06 (17)	C16—C11—C12—C13	0.7 (3)
C18—O13—C13—C14	−176.92 (16)	C12—C11—C17—O11	179.41 (18)
C18—O13—C13—C12	4.4 (3)	C16—C11—C17—O11	0.5 (3)
C6—C1—C2—C3	−0.1 (2)	C17—C11—C16—C15	177.65 (17)
C7—C1—C6—C5	179.79 (17)	C12—C11—C16—C15	−1.2 (3)
C2—C1—C6—C5	0.3 (3)	C17—C11—C12—C13	−178.19 (16)
C2—C1—C7—O1	179.85 (18)	C11—C12—C13—O13	179.34 (16)
C7—C1—C2—C3	−179.64 (16)	C11—C12—C13—C14	0.8 (2)
C6—C1—C7—O1	0.3 (3)	O13—C13—C14—C15	179.53 (16)
C1—C2—C3—C4	0.0 (2)	C12—C13—C14—O12	177.97 (16)
C1—C2—C3—O3	179.87 (16)	C12—C13—C14—C15	−1.7 (2)
O3—C3—C4—O2	0.1 (2)	O13—C13—C14—O12	−0.8 (2)
C2—C3—C4—O2	179.89 (15)	C13—C14—C15—C16	1.2 (3)
O3—C3—C4—C5	−179.95 (16)	O12—C14—C15—C16	−178.48 (17)
C2—C3—C4—C5	−0.1 (2)	C14—C15—C16—C11	0.3 (3)

Symmetry codes: (i) *x*, *y*, *z*−1; (ii) *x*, −*y*+1/2, *z*+1/2; (iii) *x*, *y*, *z*+1; (iv) *x*, −*y*+3/2, *z*−1/2; (v) *x*, −*y*+1/2, *z*−1/2; (vi) −*x*, −*y*+1, −*z*+2; (vii) *x*, −*y*+3/2, *z*+1/2; (viii) −*x*, −*y*+1, −*z*+1; (ix) −*x*+1, −*y*+1, −*z*+1.

### Hydrogen-bond geometry (Å, °)

**Table:**

*D*—H···*A*	*D*—H	H···*A*	*D*···*A*	*D*—H···*A*
O2—H1···O1^iii^	0.79 (3)	2.03 (3)	2.6951 (17)	142 (3)
O2—H1···O3	0.79 (3)	2.26 (3)	2.6619 (16)	112 (2)
O12—H14···O11^i^	0.81 (3)	2.02 (3)	2.7117 (19)	143 (3)
O12—H14···O13	0.81 (3)	2.26 (3)	2.6554 (18)	111 (2)

Symmetry codes: (iii) *x*, *y*, *z*+1; (i) *x*, *y*, *z*−1.
